# Camera trap reveals the co‐occurrence patterns of two sympatric muntjac species in southern Anhui Province, China: No spatial segregation

**DOI:** 10.1002/ece3.8307

**Published:** 2021-12-03

**Authors:** Shuaitao Deng, Jiaqi Li, Yashu Qu, Jun He, Kai Liu, Hui Xue, Peng Cui, Xiangdong Ruan, Hailong Wu

**Affiliations:** ^1^ Collaborative Innovation Center of Recovery and Reconstruction of Degraded Ecosystem in Wanjiang Basin Co‐founded by Anhui Province and Ministry of Education Provincial Key Laboratory of Biotic Environment and Ecological Safety in Anhui School of Ecology and Environment Anhui Normal University Wuhu China; ^2^ Shanghai Wildlife and Protected Natural Areas Research Center Shanghai China; ^3^ Ministry of Ecology and Environment of the People's Republic of China Nanjing Institute of Environmental Sciences Nanjing China; ^4^ College of Life Sciences Anhui Normal University Wuhu China; ^5^ Academy of Inventory and Planning National Forestry and Grassland Administration Beijing China

**Keywords:** camera trap, co‐occurrence patterns, *Muntiacus crinifrons*, *Muntiacus reevesi*, occupancy modeling

## Abstract

The competitive relationship and coexistence pattern among close related species have long been one of the hot issues in ecological research. Interspecies interactions can exert important influences on the local distribution of rare species. Black muntjac *Muntiacus crinifrons* is an endemic species to eastern China, currently restricted to limited regions. In contrast, Chinese muntjac *Muntiacus reevesi* is the most common and widespread deer in southern China. Both species co‐occur in southern Anhui and western Zhejiang Province. Little is known about the interaction of these two sympatric‐related species. In this study, to investigate the site use determinants and co‐occurrence pattern of the two sympatric muntjac species, we conducted a camera trap survey across about 250 km^2^ in mountainous area of southern Anhui Province, China. We adopted a multistep approach to incorporate habitat preferences while modeling occupancy and detection. We found that the two species did not separate along elevation gradient (range from 400 m to 1,400 m) as described in previous studies. Results of single‐species occupancy models indicated that elevation had positive effects on the site use of both species, while slope had an opposite influence on their site use. Positive effects of elevation on the site use implied that both species try to avoid human interference at low elevations. Significant negative effect of slope on the site use of black muntjac suggested that the species prefer habitat with gentle slope and avoided steep. Co‐occurrence models and species interaction factors provided evidence that the two muntjac species had an independent occupancy (*ψ* ^BM CM^ = *ψ* ^BM cm^, SIF = 1) and exhibited a positive species interaction in detection probability (*p* ^BM^ < *r* ^BM CM^). Combined with the results of previous studies, we suggested that it was fine differentiation in microhabitats and food resources utilization rather spatial or temporal segregation that allowed the two species co‐occurrence. The site use determinants revealed in our study would be useful for the habitat conservation and restoration for the rare black muntjac, and the co‐occurrence pattern of the two sympatric muntjac species could provide useful information for deep understanding of the coexistence mechanism among forest‐dwelling ungulates.

## Graphical abstract

Our study found that two sympatric‐related muntjac species, black muntjac and Chinese muntjac, did not separate along elevation gradient as described in previous studies. Co‐occurrence models and species interaction factors suggested that the two muntjacs have an independent occupancy. We suggested that it was fine differentiation in microhabitats and food resources utilization rather spatial or temporal segregation that allowed the two species co‐occurrence.

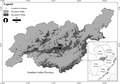

## 1 INTRODUCTION

Identifying the drivers of species coexistence is the key to understanding community assembly processes and the potential effects of environmental change (Alexander et al., 2015; Kohli et al., 2018). Morphologically similar and closely related sympatric species are expected to have high niche overlap and competition under conditions of limited resources (Schoener, 1974). In heterogeneous environments, niche segregation functions as a mechanism of coexistence among competitors, usually along spatial, temporal, and trophic axis (Amarasekare, 2003; Macandza et al., 2012; Mahendiran, 2016; Pokharel et al., 2015; Stewart et al., 2003). Of which, habitat is the major niche factor most frequently partitioned (Schoener, 1974). For example, in subtropical regions, two sympatric similar ungulates, *Tetracerus quadricornis* and *Muntiacus vaginalis*, exhibited a clear niche differentiation along environmental gradient (Pokharel et al., 2015). In Southeast Asia's tropical forests, Asian tapirs (*Tapirus indicus*) and several other sympatric ungulates showed a range of differences in response to environmental variables (Lynam et al., 2012). For example, tapirs tended to find in wetter locations and more closed forest habitats. In contrast, the other sympatric ungulates including gaur (*Bos gaurus*), red muntjac (*Muntiacus muntjak*), and sambar (*Rusa unicolor*) preferred drier areas, more open habitats, and gentler slopes. Among them, the gaur occupied lower elevations, the red muntjac occurred at higher elevations, and the sambar inhabited closer to water (Lynam et al., 2012).

Abiotic and biotic factors operate in concert on species distributional patterns, but may be effective at different scales (Chirima et al., 2013; Morin & Lechowicz, 2008). Habitat selection and interspecific competition can shape the abundance and distribution of species at local scale (Jezkova, 2020; Rosenzweig, 1981; Schoener, 1974). Habitat and interspecific relationships of rare species need special attention because biodiversity losses occurred largely through the disappearance of these species within regional assemblages (Chirima et al., 2013). Black muntjac (*Muntiacus crinifrons*) is an endemic species to eastern China and mainly occurs in mountainous areas of southern Anhui Province and western Zhejiang Province (Lu & Sheng, 1984; Sheng & Lu, 1980). Due to its limited distribution range and small population size (Ohtaishi & Gao, 1990), the species has been listed as national first‐grade protected species in China, and IUCN red directory listed it as vulnerable animal. Two other similar deer, Chinese muntjac (*Muntiacus reevesi*) and tufted deer (*Elaphodus cephalophus*), were found co‐occurrence in its distribution area (Sheng & Lu, 1980). Compared with black muntjac, both Chinese muntjac and tufted deer are common species and widespread in subtropical forests in China (Ohtaishi & Gao, 1990). Studies showed that the three sympatric deer species shared similar diets comprising tender twigs, leaves, and fruits of a diverse range of trees, shrubs, vines, and herbs (Lu & Sheng, 1984; Ou et al., 1981; Sheng & Lu, 1980). Therefore, there was potential resources exploitation competition among them. Previous studies suggested that these species segregated spatially along elevation gradient, which allowed them coexistence (Sheng, 1987; Sheng & Lu, 1980). Specifically, the black muntjac was limited to high luxuriant forest with altitude about 1,000 m, the Chinese muntjac inhabited low hilly land with altitude about 400–500 m, and the tufted deer occurred in the intermediate altitudes between the abovementioned species (Sheng, 1987; Sheng & Lu, 1980).

Because of their agile and elusive nature, few studies have conducted on the distribution dynamics and interspecific relationship of these sympatric deer. Based on the hunting harvest in winters between late 1970s and early 1980s in southern Anhui and western Zhejiang, the relative abundance (RA, expressed as percentage of the total sample deer number) was estimated 84.5%, 8.6%, and 6.9% for Chinese muntjac, black muntjac, and tufted deer, respectively (Lu & Sheng, 1984). Thus, at that time, the RA of Chinese muntjac was almost ten times that of black muntjac and the RA of black muntjac was slightly higher than that of tufted deer. However, in our recent surveys, we found that the RA of black muntjac was nearly one fifth of that of Chinese muntjac, and no images of tufted deer were captured in the surveyed regions (Liu et al., 2017). Obviously, there have been significant changes in the RAs of the three deer species over the past 40 years in these regions, and the tufted deer had vanished from these regions was possibly due to interspecific competition (Liu et al., 2017). Spatial segregation might not be the mechanism allowing these deer coexistence. The co‐occurrence patterns of black muntjac and Chinese muntjac in these regions need further survey.

Occupancy modeling is increasingly used in monitoring programs, especially when targeting elusive species (MacKenzie et al., 2002, 2006). It represents an unbiased and cost‐effective method to assess habitat use and estimate the occurrence of a species supporting robust estimates of spatial distribution status and trends (MacKenzie et al., 2002). Co‐occurrence models allow one to estimate the occurrence patterns of multiple species at a single site while explicitly fitting habitat covariates and investigating changes in occupancy and detection of one species in response to the presence of another (MacKenzie et al., 2004; Richmond et al., 2010; Steen et al., 2013). Camera trapping has proved useful for recording elusive deer with high detection efficiency (Rovero et al., 2014). In this study, we conducted a camera trap survey and applied occupancy modeling to investigate the co‐occurrence patterns of black muntjac and Chinese muntjac in southern Anhui Province. Our primary objective was to evaluate (1) those habitat variables that explained the occurrence for black muntjac and Chinese muntjac; (2) whether the two sympatric muntjac species segregated along elevation gradient; (3) whether the Chinese muntjac presence had a negative effect on the occupancy and detection probabilities of the black muntjac. Our results will aid in understanding the coexistence mechanism of similar ungulates in subtropical montane forest and be useful for conservation management of the rare black muntjac.

## 2 MATERIALS AND METHODS

### 2.1 Study area

**FIGURE 1:**
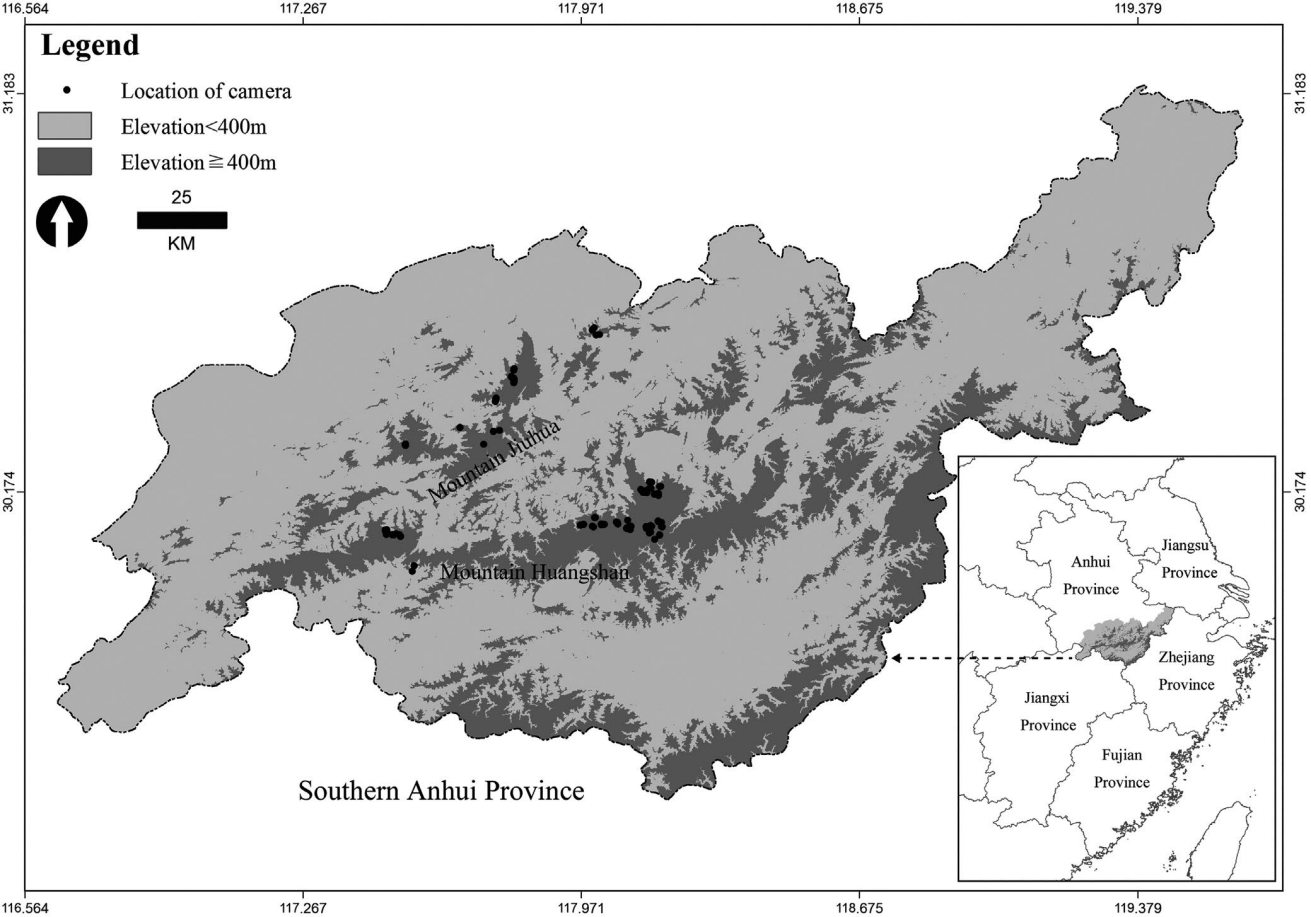
Study area and locations of camera traps in southern Anhui Province, China

The mountainous area of southern Anhui Province lies within the middle subtropical monsoon region of eastern China, bordering Zhejiang Province in the southeast, Jiangxi Province in the southwest, and the plains and hills along the Yangtze River in the north (Figure 1). The area presents subtropical humid monsoon climate, with annual precipitation of 1,200–1,700 mm, annual mean sunshine duration of 1,800–2,100 h, annual mean temperature of 15.4°C–16.3°C, and frost‐free duration of 230 days. The zonal vegetation is evergreen broad‐leaved forest. Other vegetation includes deciduous broadleaf forest, mixed coniferous and broad‐leaved forests, and bamboo stands.

The study area encompasses well‐preserved broad‐leaved forests and mixed coniferous and broad‐leaved forests, representing the main distribution areas of the black muntjac in southern Anhui Province. Our surveys conducted during summer or autumn between 2014 and 2016. In total, one hundred and eight camera traps were placed covering an area of about 250 km^2^ (coordinates 117°15′–118°17′E, 29°59′–30°40′N; Figure 1). Of which, eleven infrared cameras were not properly operational or lost during the study period, and the remaining ninety‐seven camera traps functioned effectively (Table S1). To increase the probability of target animals being photographed, in the broad‐leaved forests or mixed coniferous and broad‐leaved forests at elevations of 400–1,500 m, camera traps were often set on the tracks they are likely to pass through.

### 2.2 Camera settings and parameters

Infrared cameras used in this study were Ltl Acorn 6210 MC devices (XiaHan Creations), with images comprising 12 million pixels. Each camera was installed with a memory card (16 G SD) and 12 AA batteries (4 working batteries and 8 spare batteries), and the shooting mode was set to work continuously for 24 h. The minimum time interval between two photographs was 1 s. After each trigger, three photographs were shot continuously and a 30‐sec video was taken. Infrared cameras were mounted 0.4 m above the ground, and camera lens was adjusted so that they were generally parallel to the slope surface according to the topography. The location of each camera trap station was determined using GPS. The distance between two neighboring cameras was at least 500 m. Most infrared cameras well worked lasting for 120 days.

### 2.3 Covariates

Previous studies showed that vegetation types, canopy density, and elevation were the main environmental factors influencing the distribution of black muntjac (Lu & Sheng, 1984; Sheng & Lu, 1980). Camera traps in this study were all set up in the broad‐leaved forests, mixed coniferous and broad‐leaved forests, which was the preferred habitat of muntjac species because such kind of habitat could provide them with a rich source of food as well as good shelter (Sheng & Lu, 1980; Zheng et al., 2006). Additionally, because both species were very sensitive to human disturbance, all infrared cameras were placed in sites that were not easily accessible to humans. Therefore, vegetation types and anthropogenic factors were not included in the covariates incorporated in our models. An early study showed that black muntjac are usually found on rolling steep hills and on sheer precipices (Sheng & Lu, 1980). However, several other forest‐dwelling ungulates were found preferred gentle slope (Lynam et al., 2012). In order to evaluate the influence of slope on the occupancy of black muntjac and Chinese muntjac, in this study, slope (Slo) as well as elevation (Ele) and canopy (Can) were chosen as covariates for model detection and occupancy. The elevation of each camera site was recorded at the time of camera trap installation as mentioned above. Slope was the angle of inclination to the horizontal within 30 m of each camera trap site. Canopy density was estimated using ocular estimates of % vegetation overhead (Daubenmire, 1959).

### 2.4 Occupancy modeling

Occupancy models make use of spatial–temporal replicated data (Bailey et al., 2004) and combine the detected/undetected data of target species in each sampling period to describe maximum likelihood estimation of detection probability (*p*) and occupancy probability (*ψ*) (Stanley & Royle, 2005). To increase the detection probability for each sampling period and reduce zero inflation in the dataset, we combine 10 days into a single survey period for each camera trap site, resulting in 12 sampling occasions. For both species, detection was defined as the capture of an individual by a single camera trap. Species detection was recorded as “1” for detection and “0” for nondetection during each 10‐day period. Photographs of all individuals of the target species during one sampling period were counted as a single detection.

Our analyses were at the scale of camera trap sites rather than home ranges because no information of the size of home ranges of the two muntjacs can be available in our study area. The two muntjacs were likely to travel between camera sites during the sampling period; thus, the basic assumption of closure may be invalid (MacKenzie et al., 2006). Therefore, we interpreted occupancy (*ψ*) as the probability of site use. All covariates were first normalized to z scores before analysis and then screened for collinearity with a Spearman's correlation test, resulting no strong correlation among them (all *r* < 0.2, Appendix S2).

We adopted a two‐stage modeling approach to incorporate habitat preferences while investigating the co‐occurrence patterns (Robinson et al., 2014). In the first stage, we used single‐species occupancy models to identify relevant covariates for both species. We first defined a global model for occupancy *ψ* (Ele + Slo + Can) and held the global *ψ* constant as we developed the most suitable model for detection probability (*p*). Then carried the covariate from the best‐fitting *p* model and built a second set of candidate models to test the effects of covariates on occupancy probabilities (Royle & Nichols, 2003). To identify the best *ψ* model, we fitted all combinations of the three covariates (Ele, Slo, and Can) (Appendices S3 and S4). We evaluated candidate models and estimated parameters using PRESENCE software (Hines, 2016) to determine the covariates that best explain the occupancy and detection probabilities. We ranked candidate models using Akaike's information criterion (AIC) and excluded all models that did not converge. We considered the covariate(s) from the top‐ranked model(s) (models with ΔAIC < 2) as the most likely determinant(s) of the species’ occupancy. The relative importance of each covariate was assessed by summing AIC weights of all models that included the covariate (Burnhan & Anderson, 2002).

In the second stage, we investigated whether the presence of Chinese muntjac influences the occupancy and detection probability of black muntjac using two‐species occupancy models, while incorporating the best‐supported covariates identified in the first stage. We performed parameterization in PRESENCE software (Hines, 2016), assuming that the Chinese muntjac (CM) was the dominant species and the black muntjac (BM) the subordinate species. Because the Chinese muntjac was the most widespread muntjac in China, while the black muntjac was a rare species confined in limited regions (Ohtaishi & Gao, 1990), and the former had a much higher RA in the study area than that of the latter (Liu et al., 2017; Lu & Sheng, 1984). We estimated the following probabilities: *ψ* ^CM^ (occupancy probability of the dominant species, i.e., the Chinese muntjac), *ψ* ^BM CM^ (occupancy probability of the subordinate species, i.e., the black muntjac, when the dominant is present), and *ψ* ^BM cm^ (occupancy probability of the subordinate species in the absence of the dominant species). We built a set of a priori models that assumed that the presence of the dominant species influenced the subordinate (*ψ* ^CM^ ≠ *ψ* ^BM CM^ ≠ *ψ* ^BM cm^) and constrained models where the occupancy of the subordinate was independent of the presence of the dominant species (*ψ* ^CM^ ≠ *ψ* ^BM CM^ = *ψ* ^BM cm^). The SIF (species interaction factor) is a ratio of how likely the two species are to co‐occur compared to what would be expected under a hypothesis of independence (Richmond et al., 2010). If SIF = 1, the species co‐occur or are detected together about as frequently as expected under the null hypothesis of independence, while SIF < 1 suggests avoidance and SIF > 1 indicates co‐occurrence. We used AIC to rank candidate models. To infer about the co‐occurrence patterns, we considered the estimated parameters of the top‐ranked models (ΔAIC < 2) and the calculated SIF.

For detection probability, we estimated the following parameters: *p* ^CM^ (probability of detecting the dominant species, given the absence of the subordinate), *p* ^BM^ (probability of detecting the subordinate, given the absence of the dominant), *r* ^CM^ (probability of detecting the dominant, given both are present), *r* ^BM CM^ (probability of detecting the subordinate, given both are present and the dominant is detected), and *r* ^BM cm^ (probability of detecting the subordinate species, given both are present and the dominant is not detected). We built a set of a priori models assuming that the detection probabilities of each species were independent of the presence or detection of the other (*p* ^CM^ = *r* ^CM^ and *p* ^BM^ = *r* ^BM CM^ = *r* ^BM cm^) and models where each species was influenced by the presence and detection of the other (*p* ^CM^ ≠ *r* ^CM^ and *p* ^BM^ ≠ *r* ^BM CM^ ≠ *r* ^BM cm^).

## 3 RESULTS

Totally, the Chinese muntjac was captured at 74 camera trap sites and its naïve occupancy was 0.76 (74/97) (Appendix S1). In contrast, the black muntjac was recorded by 38 camera traps and its naïve occupancy was 0.39 (38/97) (Appendix S1). Both species were found inhabiting throughout the elevation range of 400 m−1,400 m in the surveyed regions. In addition, 82% (31/38) of the camera sites recording black muntjac also found Chinese muntjac (Appendix S1), indicating a high degree of habitat overlap between the two species.

### 3.1 Single‐species occupancy models

The detection of Chinese muntjac was best explained by model of slope (AICw = 0.47), while the detection of black muntjac was best illustrated by model of elevation (AICw = 0.28) (Table 1). Model average of the beta coefficients for covariates included in the top‐ranking single‐species models indicated that slope had a well‐supported negative effect on the detection of Chinese muntjac (*β* ± SE = −0.23 ± 0.09), while elevation exerted a strong negative effect on the detection of black muntjac (*β* ± SE = −0.55 ± 0.23) (Table 2).

**TABLE 1:** Summed single‐species occupancy models selection results indicating the role of covariates in determining probabilities of Chinese muntjac (CM) and black muntjac (BM) detection and site use, showing estimated occupancy probability (*ψ*) and detectability (*p*) for the models with ∆AIC <2

Species	Model	AIC	ΔAIC	AICw	ML	*K*	LL	*p/ψ*
CM	*Detection model selection*							
	*ψ*(Slo + Ele + Can), *p*(Slo)	1143.69	0	0.47	1.00	6	1131.69	.268
	*ψ*(Slo+Ele + Can), *p*(Slo + Can)	1145.46	1.77	0.20	0.41	7	1131.46	.267
	*ψ*(Slo+Ele + Can), *p*(Ele + Slo)	1145.61	1.92	0.18	0.38	7	1131.61	.268
	*Occupancy model selection*							
	*ψ*(Ele + Can), *p*(Slo)	1142.75	0	0.21	1.00	5	1132.75	.782
	*ψ*(Can), *p*(Slo)	1142.92	0.17	0.19	0.92	4	1134.92	.781
	*ψ*(Ele), *p*(Slo)	1143.56	0.81	0.14	0.67	4	1135.56	.783
	*ψ*(Slo + Ele + Can), *p*(Slo)	1143.69	0.94	0.13	0.63	6	1131.69	.784
	*ψ*(Slo + Can), *p*(Slo)	1144.11	1.36	0.10	0.51	5	1134.11	.783
	*ψ*(.), *p*(Slo)	1144.27	1.52	0.10	0.47	3	1138.27	.782
	*ψ*(Slo + Ele), *p*(Slo)	1144.57	1.82	0.08	0.40	5	1134.57	.785
BM	*Detection model selection*							
	*ψ*(Slo + Ele + Can), *p*(Ele)	516.96	0	0.28	1.00	6	504.96	.133
	*ψ*(Slo + Ele + Can), *p*(Ele + Slo)	517.68	0.72	0.20	0.70	7	503.68	.128
	*ψ*(Slo + Ele+Can), *p*(Can + Ele)	517.97	1.01	0.17	0.60	7	503.97	.133
	*ψ*(Slo + Ele + Can), *p*(Slo + Ele + Can)	518.15	1.19	0.16	0.55	8	502.15	.126
	*Occupancy model selection*							
	*ψ*(Slo), *p*(Ele)	514.98	0	0.27	1.00	4	506.98	.474
	*ψ*(Slo + Ele), *p*(Ele)	515.07	0.09	0.26	0.96	5	505.07	.490
	*ψ*(Ele), *p*(Ele)	516.72	1.74	0.12	0.42	4	508.72	.507
	*ψ*(Slo + Can), *p*(Ele)	516.87	1.89	0.11	0.39	5	506.87	.475
	*ψ*(Slo + Ele + Can), *p*(Ele)	516.96	1.98	0.10	0.37	6	504.96	.492

**TABLE 2:** Estimates of model averaged β coefficient values and standard error (SE) for covariates included in the well‐supported single‐species occupancy models (ΔAIC < 2) for Chinese muntjac (CM) and black muntjac (BM)

		CM	BM
	Covariates	*β* coefficient ± SE	*β* coefficient ± SE
Detection	Can	0.04 ± 0.09	0.20 ± 0.18
	Slo	−**0.23** ± **0.09**	−0.23 ± 0.20
	Ele	−0.03 ± 0.11	−**0.55** ± **0.23**
Occupancy	Can	**0.53** ± **0.30**	0.10 ± 0.30
	Slo	0.29 ± 0.31	−**0.57** ± **0.29**
	Ele	**0.62** ± **0.40**	**0.64** ± **0.46**

Note

Bold indicates a well‐supported effect.

The occupancy of Chinese muntjac was best explained by model comprising canopy and elevation (AICw = 0.21), and that of black muntjac was best illustrated by model of slope (AICw = 0.27) (Table 1). Summed AICw indicated that canopy (0.63) was the most important covariate accounting for the occupancy of Chinese muntjac, while slope (0.75) was the first determinant of black muntjac’ occupancy. Elevation had a similar effect on the occupancy of both species, with summed AICw of 0.56 for Chinese muntjac and 0.52 for black muntjac (Appendix S5). Model average of *β* value indicated that canopy and elevation had significant positive effects on the occupancy of Chinese muntjac (*β* ± SE = 0.53 ± 0.30 and 0.62 ± 0.40, respectively). For black muntjac, elevation also had a well‐supported positive effect on the site use (*β* ± SE = 0.64 ± 0.46); however, slope exhibited a strong negative influence on it (*β* ± SE = −0.57 ± 0.29) (Table 2). Additionally, slope had positive effects on the occupancy of Chinese muntjac and canopy also had positive effects on that of black muntjac, but neither was significant (Table 2).

### 3.2 Co‐occurrence occupancy models

There was no evidence that the presence of Chinese muntjac influenced the probability of site use of black muntjac (*ψ* ^BM CM^ = *ψ* ^BM cm^). After accounting for covariate (slope), there was no change in the occupancy probability of black muntjac with the presence of Chinese muntjac (Table 3). The species interaction factor also suggested independence between the two muntjac species (SIF = 1; Table 4).

**TABLE 3:** Two‐species occupancy and detection models used to evaluate the role of interspecific interactions between Chinese muntjac (CM) and black muntjac (BM)

	Model	AIC	ΔAIC	AICw	ML	*K*	LL
Occupancy	***ψ* ^CM^(Ele** + **Can)** ≠ ***ψ* ^BM CM^** = ***ψ* ^BM cm^(Slo), *p* ^CM^** ≠ ***r* ^CM^ (Slo), *p* ^BM^** ≠ ***r* ^BM CM^** ≠ ***r* ^BM cm^ (Ele)**	**1637.6**	**0**	**0.76**	**1.00**	**15**	**1607.6**
	*ψ* ^CM^(Ele + Can) ≠ *ψ* ^BM CM^ ≠ *ψ* ^BM cm^(Slo), *p* ^CM^ ≠ *r* ^CM^ (Slo), *p* ^BM^ ≠ *r* ^BM CM^ ≠ *r* ^BM cm^ (Ele)	1641.1	3.5	0.13	0.17	17	1607.1
	*ψ* ^CM^(.) ≠ *ψ* ^BM CM^ = *ψ* ^BM cm^(.),*p* ^CM^ ≠ *r* ^CM^ (Slo), *p* ^BM^ ≠ *r* ^BM CM^ ≠ *r* ^BM cm^ (Ele)	1642.09	4.49	0.08	0.11	12	1618.09
	*ψ* ^CM^(.) ≠ *ψ* ^BM CM^ ≠ *ψ* ^BM cm^(.), *p* ^CM^ ≠ *r* ^CM^ (Slo), *p* ^BM^ ≠ *r* ^BM CM^ ≠ *r* ^BM cm^ (Ele)	1644.07	6.47	0.03	0.04	13	1618.07
Detection	***ψ* ^CM^(Ele** + **Can)** ≠ ***ψ* ^BM CM^** = ***ψ* ^BM cm^(Slo), *p* ^CM^** ≠ ***r* ^CM^ (.), *p* ^BM^** ≠ ***r* ^BM CM^** ≠ ***r* ^BM cm^ (.)**	**1636.93**	**0**	**0.58**	**1.00**	**10**	**1616.93**
	***ψ* ^CM^(Ele** + **Can)** ≠ ***ψ* ^BM CM^** = ***ψ* ^BM cm^(Slo), *p* ^CM^** ≠ ***r* ^CM^ (Slo), *p* ^BM^** ≠ ***r* ^BM CM^** ≠ ***r* ^BM cm^ (Ele)**	**1637.6**	**0.67**	**0.42**	**0.72**	**15**	**1607.6**
	*ψ* ^CM^(Ele + Can) ≠ *ψ* ^BM CM^ = *ψ* ^BM cm^(Slo), *p* ^CM^ ≠ *r* ^CM^ (Slo), *p* ^BM^ ≠ *r* ^BM CM^ ≠ *r* ^BM cm^ (Ele)	1651.31	14.38	0.00	0.00	9	1633.31
	*ψ* ^CM^(Ele + Can) ≠ *ψ* ^BM CM^ = *ψ* ^BM cm^(Slo), *p* ^CM^ ≠ *r* ^CM^ (.), *p* ^BM^ ≠ *r* ^BM CM^ ≠ *r* ^BM cm^ (.)	1655.87	18.94	0.00	0.00	7	1641.87

Note

Models with ΔAIC < 2 are marked in bold.

**TABLE 4:** Estimates of model averaged occupancy probability, detectability, species interaction (SIF) and standard error (SE) included in the well‐supported two‐species occupancy models (ΔAIC < 2)

	model	*ψ* ^CM^ ± SE	*ψ* ^BM CM^ ± SE	*ψ* ^BM cm^ ± SE	SIF ± SE	
occupancy	***ψ* ^CM^(Ele **+ **Can)** ≠ ***ψ* ^BM CM^** = ***ψ* ^BM cm^(Slo)**, ***p* ^CM^** ≠ ***r* ^CM^ (Slo), *p* ^BM^** ≠ ***r* ^BM CM^** ≠ ***r* ^BM cm^ (Ele)**	0.78 ± 0.07	0.48 ± 0.09	0.48 ± 0.09	1.00 ± 0.00	
	Model	*p* ^CM^ ± SE	*r* ^CM^ ± SE	*p* ^BM^ ± SE	*r* ^BM CM^ ± SE	*r* ^BM cm^ ± SE
detection	***ψ* ^CM^(Ele** + **Can)** ≠ ***ψ* ^BM CM^** = ***ψ* ^BM cm^(Slo), *p* ^CM^** ≠ ***r* ^CM^ (.), *p* ^BM^** ≠ ***r* ^BM CM^** ≠ ***r* ^BM cm^ (.)**	0.18 ± 0.03	0.34 ± 0.02	0.11 ± 0.04	0.20 ± 0.04	0.08 ± 0.02
	***ψ* ^CM^(Ele** + **Can)** ≠ ***ψ* ^BM CM^** = ***ψ* ^BM cm^(Slo), *p* ^CM^** ≠ ***r* ^CM^ (Slo), *p* ^BM^** ≠ ***r* ^BM CM^** ≠ ***r* ^BM cm^ (Ele)**	0.25 ± 0.04	0.28 ± 0.04	0.11 ± 0.05	0.27 ± 0.07	0.09 ± 0.02

As for detection, of the two top‐ranked models, one was accounting for covariates and the other was not (Table 3). Both models indicated that the presence of Chinese muntjac had positive influences on the detection probability of black muntjac (*p* ^BM^ < *r* ^BM CM^; Table 4). This positive species interaction may be explained by common preference of the two species for vegetation types (broad‐leaved forests, mixed coniferous, and broad‐leaved forests) as mentioned above.

## 4 DISCUSSION

In this study, we evaluated the distribution status, determinants of site use, and co‐occurrence patterns of two sympatric muntjacs in southern Anhui Province.

As was expected, the Chinese muntjac found across most of the camera trap sites (74/97), while the black muntjac was recorded in less than half of the sites (38/97) and exhibited a relative low occupancy (Appendix S1). We also confirmed the result of our earlier study (Liu et al., 2017) that the RA of tufted deer might be extremely low and even disappear from this area, because no image of the species was recorded by any of the camera traps in this study. The ratio of the RA of the black muntjac and the Chinese muntjac (data not shown) was similar to the value reported by Liu et al. (2017), which was higher than that (about 1:10) obtained by Lu and Sheng (1984) during 1978–1979. These results indicated that, in the last few decades, the RA of the black muntjac has increased significantly while that of the tufted deer has decreased sharply in this area. Previous studies showed that the feeding habits of the black muntjac and the sympatric tufted deer were similar, with the major plant species in their diets being identical (Lu & Sheng, 1984; Ou et al., 1981). Thus, the changes in the RAs of the two sympatric species might be resulted from potential interspecific competition.

Results from two‐species occupancy models indicated an independent occurrence pattern of the two muntjacs in the study area (Tables 3 and 4). Habitat partitioning is often suggested the primary mechanism for coexistence between similar species (Schoener, 1974; van Beest et al., 2014), which was also verified in several forest‐dwelling ungulates (Lynam et al., 2012; Pokharel et al., 2015). However, we found that the two muntjacs did not separate along elevation gradient because their habitats highly overlapped, which disproved previous hypothesis that the two sympatric species were spatially separated (Sheng, 1987; Sheng & Lu, 1980). These results suggested that spatial segregation be not the mechanism allowing the two muntjac species co‐occurrence in the study area. Additionally, previous studies showed that the two species were strictly diurnal animal and shared a similar daily activity rhythm (Liu et al., 2017; Zhang et al., 2012), implying that the two muntjac species might also not be differentiated in temporal niche.

We suggested several potential mechanisms that allowed the two animals to coexist. Firstly, model average of the beta coefficients confirmed that both muntjacs tended to live at higher altitudes (Table 2), which could be interpreted as both species trying to avoid human interference. However, slope had an opposite influence on the site use of the two species (Table 2). It had a significant negative effect on the occupancy of black muntjac, indicating that species preferred to choose habitat with gentle slope, as was similar to the terrain preference of some other forest‐dwelling ungulates (Lynam et al., 2012; Pokharel et al., 2015), rather than sheer precipices and overhanging rocks described in previous studies (Sheng & Lu, 1980). In contrast, slope had slight positive effects on the site use of Chinese muntjac (Table2), suggesting that the species could adapt to complex and varied terrains, which might allow the species to mitigate potential interspecific competition by using diverse microhabitats. Study on Taiwan subspecies (*M*. *r*. *micrurus*) also confirmed that the species could occupy nearly all of the broad‐leaved forests and use nearly all of the microhabitats in the study area (Mccullough et al., 2000). Thus, we suggested that the difference in microhabitats utilization might be one of the important factors allowing the two species to coexist in the study area.

Secondly, studies on the food habit showed that though the Chinese muntjac shared a range of food items similar with that of black muntjac, there was distinct difference in the parts of plants consumed by them (Ou et al., 1981; Sheng & Lu, 1980; Lu & Sheng, 1984). For example, in winter, Chinese muntjac preferred foraging fallen fruits and seeds, while the black muntjac feed predominantly upon tender twigs and leaves of many evergreens and had less preference for fruit and seeds (Lu & Sheng, 1984). Thirdly, there was a great difference in body size between the two species. The black muntjac adults measured 62.2–78 cm high at the shoulder compared to that of 40.6–48.4 cm for the Chinese munjtac (Ma et al., 1986), which allowed the former species reaching food in tall plants that the latter could not approach. Finally, the Chinese muntjac could use a wide variety of foods depending upon what was available, even grass when no other food resources could be used (Jackson & Chapman, 1977). In contrast, studies have shown that the black muntjac never chose grasses (Sheng & Lu, 1980; Lu & Sheng, 1984). In conclusion, we suggested that the fine differentiation in feeding habit and the difference in the ability to acquire and/or utilize resources might serve to mitigate interspecies competition caused by habitat overlap, allowing the two species co‐occurrence.

However, habitat selection and partitioning among similar species was suggested density‐dependent (Van Beest et al., 2014). Therefore, further studies should focus on the relationship between the coexistence patterns and population density/relative abundance of the two species. Additionally, though occupancy models are increasingly applied to data from wildlife camera‐trap surveys to estimate distribution, habitat use, and patterns of species co‐occurrence of unmarked animals (Burton et al., 2015; Estevo et al., 2017), the interpretation of co‐occurrence patterns is inherently difficult (Barros et al., 2020). Several studies have demonstrated that detection frequency and estimates of detection probability and occupancy were sensitive to movement speed (or the magnitude of animal movement) (Neilson et al., 2018; Stewart et al., 2018), especially for animal occupied large‐ and medium‐sized home ranges with low population density (Neilson et al., 2018), whereas occupancy estimates for scenarios with small home ranges closely matched the simulated asymptotic PAO (proportion of area occupied) (Neilson et al., 2018). In this study, both muntjac species are small‐sized deer dwelling mountainous forest, in contrast to the large‐ and medium‐sized ungulates that inhabit the plains, they should have relative small home range and similar movement speed. Therefore, we believe that our sampling scenario and analytical framework should match the ecology of the target species.

## CONFLICT OF INTEREST

The authors declare that they have no conflict interests.

## AUTHOR CONTRIBUTIONS

**Deng Shuaitao:** Data curation (lead); formal analysis (equal); investigation (equal); methodology (lead); writing–original draft (lead). **Jiaqi Li:** data curation (equal); formal analysis (equal); investigation (supporting); methodology (equal); writing–review and editing (equal). **Yashu Qu:** Data curation (equal); investigation (equal). **Jun He:** Data curation (equal); investigation (equal); methodology (equal). **Kai Liu:** Data curation (equal); investigation (equal); visualization (equal). **Hui Xue:** Formal analysis (equal); visualization (equal). **Peng Cui:** Formal analysis (equal); methodology (equal). **Xiangdong Ruan:** Conceptualization (equal); funding acquisition (equal); resources (equal). **Hailong Wu:** Conceptualization (lead); formal analysis (lead); funding acquisition (lead); supervision (lead); writing–review and editing (lead).

## Supporting information

Appendix S1‐S5

## Data Availability

All raw data are available on FigShare (https://doi.org/10.6084/m9.figshare.14827788).
