# Supplementary material for: Camera trap reveals the co‐occurrence patterns of two sympatric muntjac species in southern Anhui Province, China: No spatial segregation
Source: Ecol Evol. 2021 Dec 3;11(24):17801–9. doi: 10.1002/ece3.8307 (PMC8717271; doi:10.1002/ece3.8307)
Supplement: Supplementary file 1 — Appendix S1‐S5 [file ECE3-11-17801-s001.docx]

APPENDIXS

Appendix S1. Information of camera sites used in this study and the sites where target animals captured by camera trpas. The mark “√” represent where the target animals were photoSlophed by camera trap.

| samples | GeoSlophical coordinate | Elevation (m) | Slodient | Canopy density | Black muntjac | Reeve’s muntjac |
| --- | --- | --- | --- | --- | --- | --- |
| 1 | E 117°28′13″, N 30°01′35″ | 814 | 15 | 0.5 | √ | √ |
| 2 | E 117°28′01″, N 30°01′16″ | 714 | 30 | 0.3 |  |  |
| 3 | E 117°28′30″, N 30°01′38″ | 684 | 20 | 0.6 | √ |  |
| 4 | E 117°27′15″, N 30°05′48″ | 714 | 20 | 0.5 |  |  |
| 5 | E 117°28′36″, N 30°04′43″ | 615 | 25 | 0.3 |  |  |
| 6 | E 117°26′55″, N 30°03′52″ | 1121 | 30 | 0.4 |  | √ |
| 7 | E 117°28′07″, N 30°01′29″ | 610 | 40 | 0.3 |  | √ |
| 8 | E 117°29′01″, N 30°03′51″ | 822 | 20 | 0.6 |  | √ |
| 9 | E 117°27′58″, N 30°01′08″ | 674 | 30 | 0.4 |  |  |
| 10 | E 117°27′10″, N 30°05′03″ | 892 | 45 | 0.7 |  |  |
| 11 | E 117°28′46″, N 30°04′46″ | 550 | 15 | 0.8 |  | √ |
| 12 | E 117°30′49″, N 30°03′46″ | 721 | 30 | 0.6 | √ |  |
| 13 | E 117°28′42″, N 30°04′02″ | 612 | 15 | 0.8 | √ | √ |
| 14 | E 117°29′42″, N 30°03′59″ | 713 | 70 | 0.8 | √ | √ |
| 15 | E 117°29′03″, N 30°03′48″ | 732 | 30 | 0.7 | √ |  |
| 16 | E 117°30′54″, N 30°03′47″ | 733 | 20 | 0.7 | √ |  |
| 17 | E 117°28′33″, N 30°04′43″ | 572 | 30 | 0.75 |  | √ |
| 18 | E 117°29′37″, N 30°04′01″ | 823 | 30 | 0.5 | √ | √ |
| 19 | E 117°29′51″, N 30°04′01″ | 551 | 70 | 0.6 | √ | √ |
| 20 | E 117°30′12″, N 30°04′11″ | 596 | 60 | 0.6 |  | √ |
| 21 | E 117°30′43″, N 30°03′59″ | 686 | 20 | 0.7 |  | √ |
| 22 | E 117°28′48″, N 30°04′20″ | 581 | 15 | 0.8 | √ | √ |
| 23 | E 117°28′42″, N 30°04′32″ | 730 | 20 | 0.75 | √ | √ |
| 24 | E 118°10′01″, N 30°10′16″ | 771 | 20 | 0.5 | √ | √ |
| 25 | E 118°10′11″, N 30°11′21″ | 620 | 0 | 0.4 | √ | √ |
| 26 | E 118°08′34″, N 30°10′56″ | 720 | 0 | 0.8 | √ | √ |
| 27 | E 118°10′24″, N 30°11′21″ | 612 | 20 | 0.4 | √ | √ |
| 28 | E 118°08′04″, N 30°10′42″ | 824 | 20 | 0.6 | √ | √ |
| 29 | E 118°07′55″, N 30°10′41″ | 883 | 0 | 0.7 | √ | √ |
| 30 | E 118°08′05″, N 30°10′42″ | 794 | 0 | 0.4 | √ | √ |
| 31 | E 118°08′17″, N 30°10′22″ | 716 | 10 | 0.5 | √ |  |
| 32 | E 118°09′24″, N 30°10′09″ | 772 | 10 | 0.4 | √ | √ |
| 33 | E 118°10′01″, N 30°09′57″ | 771 | 20 | 0.5 |  | √ |
| 34 | E 118°07′29″, N 30°10′43″ | 923 | 10 | 0.6 | √ | √ |
| 35 | E 118°07′22″, N 30°10′55″ | 934 | 10 | 0.8 |  | √ |
| 36 | E 118°07′55″, N 30°10′25″ | 923 | 40 | 0.6 |  | √ |
| 37 | E 118°08′53″, N 30°11′05″ | 663 | 25 | 0.4 |  | √ |
| 38 | E 118°07′42″, N 30°10′25″ | 921 | 0 | 0.8 | √ | √ |
| 39 | E 118°09′47″, N 30°10′13″ | 747 | 30 | 0.6 |  | √ |
| 40 | E 118°08′16″, N 30°05′13″ | 793 | 60 | 0.8 |  | √ |
| 41 | E 118°08′40″, N 30°04′16″ | 778 | 50 | 0.4 |  |  |
| 42 | E 118°08′14″, N 30°04′59″ | 904 | 40 | 0.4 |  | √ |
| 43 | E 118°08′29″, N 30°04′54″ | 806 | 25 | 0.7 |  | √ |
| 44 | E 118°10′29″, N 30°05′13″ | 764 | 30 | 0.6 |  | √ |
| 45 | E 118°08′56″, N 30°05′14″ | 712 | 25 | 0.4 |  |  |
| 46 | E 118°08′59″, N 30°05′19″ | 783 | 30 | 0.8 | √ | √ |
| 47 | E 118°10′02″, N 30°05′59″ | 821 | 30 | 0.8 |  | √ |
| 48 | E 118°09′58″, N 30°06′05″ | 800 | 30 | 0.6 |  |  |
| 49 | E 118°08′51″, N 30°05′27″ | 752 | 40 | 0.6 |  | √ |
| 50 | E 118°08′23″, N 30°05′03″ | 790 | 60 | 0.5 |  | √ |
| 51 | E 118°08′23″, N 30°05′12″ | 712 | 40 | 0.6 |  | √ |
| 52 | E 118°10′37″, N 30°05′10″ | 687 | 40 | 0.7 |  | √ |
| 53 | E 118°10′11″, N 30°05′57″ | 684 | 40 | 0.7 |  | √ |
| 54 | E 118°08′49″, N 30°04′22″ | 780 | 40 | 0.6 |  | √ |
| 55 | E 118°10′13″, N 30°05′58″ | 685 | 50 | 0.5 |  | √ |
| 56 | E 118°10′29″, N 30°05′51″ | 702 | 30 | 0.4 |  | √ |
| 57 | E 117°47′57″, N 30°27′39″ | 900 | 25 | 0.75 | √ | √ |
| 58 | E 117°47′39″, N 30°28′03″ | 804 | 15 | 0.85 | √ | √ |
| 59 | E 117°48′08″, N 30°29′14″ | 690 | 30 | 0.7 |  | √ |
| 60 | E 117°48′03″, N 30°27′48″ | 830 | 15 | 0.9 | √ | √ |
| 61 | E 117°48′03″, N 30°27′07″ | 857 | 0 | 0.85 |  | √ |
| 62 | E 117°47′59″, N 30°26′40″ | 890 | 20 | 0.7 |  | √ |
| 63 | E 117°48′08″, N 30°27′41″ | 718 | 35 | 0.7 |  | √ |
| 64 | E 117°48′05″, N 30°28′59″ | 683 | 30 | 0.7 |  | √ |
| 65 | E 117°56′16″, N 30°04′46″ | 1153 | 25 | 0.7 |  | √ |
| 66 | E 118°05′10″, N 30°04′57″ | 1063 | 20 | 0.6 | √ | √ |
| 67 | E 118°01′21″, N 30°05′32″ | 750 | 30 | 0.8 |  | √ |
| 68 | E 117°59′59″, N 30°05′19″ | 880 | 20 | 0.8 |  |  |
| 69 | E 118°03′51″, N 30°05′51″ | 755 | 20 | 0.8 | √ | √ |
| 70 | E 118°00′18″, N 30°06′36″ | 780 | 15 | 0.7 | √ | √ |
| 71 | E 117°58′10″, N 30°05′28″ | 813 | 20 | 0.6 | √ | √ |
| 72 | E 117°58′42″, N 30°05′35″ | 1010 | 20 | 0.5 | √ |  |
| 73 | E 118°00′27″, N 30°06′38″ | 760 | 20 | 0.6 |  |  |
| 74 | E 118°05′20″, N 30°04′58″ | 1017 | 20 | 0.7 |  | √ |
| 75 | E 118°01′50″, N 30°05′36″ | 509 | 20 | 0.7 |  | √ |
| 76 | E 118°01′21″, N 30°05′38″ | 850 | 25 | 0.7 |  |  |
| 77 | E 118°05′20″, N 30°06′13″ | 493 | 5 | 0.7 |  | √ |
| 78 | E 118°00′11″, N 30°05′10″ | 1060 | 5 | 0.7 | √ | √ |
| 79 | E 118°05′13″, N 30°05′05″ | 711 | 15 | 0.9 |  | √ |
| 80 | E 118°05′41″, N 30°05′15″ | 1130 | 15 | 0.4 | √ | √ |
| 81 | E 118°03′52″, N 30°05′40″ | 888 | 25 | 0.8 |  | √ |
| 82 | E 118°03′39″, N 30°05′55″ | 935 | 10 | 0.8 |  | √ |
| 83 | E 117°45′53″, N 30°19′51″ | 628 | 40 | 0.6 |  | √ |
| 84 | E 117°31′37″, N 30°17′34″ | 644 | 0 | 0.8 |  |  |
| 85 | E 117°45′21″, N 30°24′28″ | 521 | 0 | 0.4 |  |  |
| 86 | E 117°31′39″, N 30°17′50″ | 597 | 10 | 0.3 | √ | √ |
| 87 | E 117°43′31″, N 30°17′43″ | 542 | 50 | 0.5 |  |  |
| 88 | E 117°45′20″, N 30°24′26″ | 430 | 40 | 0.7 | √ | √ |
| 89 | E 117°31′37″, N 30°17′34″ | 729 | 40 | 0.6 |  | √ |
| 90 | E 117°39′53″, N 30°20′18″ | 651 | 0 | 0.4 |  | √ |
| 91 | E 117°45′15″, N 30°24′16″ | 420 | 0 | 0.8 |  |  |
| 92 | E 117°31′35″, N 30°17′46″ | 670 | 40 | 0.7 |  | √ |
| 93 | E 117°45′24″, N 30°24′45″ | 558 | 30 | 0.8 | √ |  |
| 94 | E 117°44′59″, N 30°19′41″ | 774 | 60 | 0.5 | √ | √ |
| 95 | E 118°00′32″, N 30°34′21″ | 452 | 30 | 0.4 | √ | √ |
| 96 | E 118°01′03″, N 30°34′23″ | 448 | 15 | 0.4 |  |  |
| 97 | E 117°59′58″, N 30°35′07″ | 461 | 45 | 0.7 |  | √ |

Appendix S2. Results of pairwise Spearman’s correlation of all three covariates. Covariate abbreviations are elevation (Ele), slope (slo) and canopy density (Can).

|  | Ele | Slo | Can |
| --- | --- | --- | --- |
| Ele | 1.00 | -0.13 | 0.14 |
| Slo | -0.13 | 1.00 | -0.14 |
| Can | 0.14 | -0.14 | 1.00 |

Appendix S3. Summary of single-species occupancy models selection results indicating the role of covariates in determining probabilities of Chinese muntjac detection and site use.

| Model | AIC | ΔAIC | AICw | ML | K | LL |
| --- | --- | --- | --- | --- | --- | --- |
| **Detection model selection** |  |  |  |  |  |  |
| ***ψ*(Slo+Ele+Can),*p*(Slo)** | **1143.69** | **0** | **0.47** | **1.00** | **6** | **1131.69** |
| ***ψ*(Slo+Ele+Can),*p*(Slo+Can)** | **1145.46** | **1.77** | **0.20** | **0.41** | **7** | **1131.46** |
| ***ψ*(Slo+Ele+Can),*p*(Ele+Slo)** | **1145.61** | **1.92** | **0.18** | **0.38** | **7** | **1131.61** |
| *ψ*(Slo+Ele+Can),*p*(Slo+Ele+Can) | 1147.37 | 3.68 | 0.08 | 0.16 | 8 | 1131.37 |
| *ψ*(Slo+Ele+Can),*p*(.) | 1148.85 | 5.16 | 0.04 | 0.08 | 5 | 1138.85 |
| *ψ*(Slo+Ele+Can),*p*(Can) | 1150.43 | 6.74 | 0.02 | 0.03 | 6 | 1138.43 |
| *ψ*(Slo+Ele+Can),*p*(Ele) | 1150.7 | 7.01 | 0.01 | 0.03 | 6 | 1138.7 |
| *ψ*(Slo+Ele+Can),*p*(Can+Ele) | 1152.3 | 8.61 | 0.01 | 0.01 | 7 | 1138.3 |
| **Occupancy model selection** |  |  |  |  |  |  |
| ***ψ*(Ele+Can),*p*(Slo)** | **1142.75** | **0** | **0.21** | **1.00** | **5** | **1132.75** |
| ***ψ*(Can),*p*(Slo)** | **1142.92** | **0.17** | **0.19** | **0.92** | **4** | **1134.92** |
| ***ψ*(Ele),*p*(Slo)** | **1143.56** | **0.81** | **0.14** | **0.67** | **4** | **1135.56** |
| ***ψ*(Slo+Ele+Can),*p*(Slo)** | **1143.69** | **0.94** | **0.13** | **0.63** | **6** | **1131.69** |
| ***ψ*(Slo+Can),*p*(Slo)** | **1144.11** | **1.36** | **0.10** | **0.51** | **5** | **1134.11** |
| ***ψ*(.),*p*(Slo)** | **1144.27** | **1.52** | **0.10** | **0.47** | **3** | **1138.27** |
| ***ψ*(Slo+Ele),*p*(Slo)** | **1144.57** | **1.82** | **0.08** | **0.40** | **5** | **1134.57** |
| *ψ*(Slo),*p*(Slo) | 1145.59 | 2.84 | 0.05 | 0.24 | 4 | 1137.59 |

Appendix S4. Summary of single-species occupancy models selection results indicating the role of covariates in determining probabilities of black muntjac detection and site use.

| Model | AIC | ΔAIC | AICw | ML | K | LL |
| --- | --- | --- | --- | --- | --- | --- |
| **Detection model selection** |  |  |  |  |  |  |
| ***ψ*(Slo+Ele+Can),*p*(Ele)** | **516.96** | **0** | **0.28** | **1.00** | **6** | **504.96** |
| ***ψ*(Slo+Ele+Can),*p*(Ele+Slo)** | **517.68** | **0.72** | **0.20** | **0.70** | **7** | **503.68** |
| ***ψ*(Slo+Ele+Can),*p*(Can+Ele)** | **517.97** | **1.01** | **0.17** | **0.60** | **7** | **503.97** |
| ***ψ*(Slo+Ele+Can),*p*(Slo+Ele+Can)** | **518.15** | **1.19** | **0.16** | **0.55** | **8** | **502.15** |
| *ψ*(Slo+Ele+Can),*p*(.) | 519.36 | 2.4 | 0.09 | 0.30 | 5 | 509.36 |
| *ψ*(Slo+Ele+Can),*p*(Can) | 520.24 | 3.28 | 0.05 | 0.19 | 6 | 508.24 |
| *ψ*(Slo+Ele+Can),*p*(Slo) | 521.36 | 4.4 | 0.03 | 0.11 | 6 | 509.36 |
| *ψ*(Slo+Ele+Can),*p*(Can+Slo) | 522.21 | 5.25 | 0.02 | 0.07 | 7 | 508.21 |
| **Occupancy model selection** |  |  |  |  |  |  |
| ***ψ*(Slo),*p*(Ele)** | **514.98** | **0** | **0.27** | **1.00** | **4** | **506.98** |
| ***ψ*(Slo+Ele),*p*(Ele)** | **515.07** | **0.09** | **0.26** | **0.96** | **5** | **505.07** |
| ***ψ*(Ele),*p*(Ele)** | **516.72** | **1.74** | **0.12** | **0.42** | **4** | **508.72** |
| ***ψ*(Slo+Can),*p*(Ele)** | **516.87** | **1.89** | **0.11** | **0.39** | **5** | **506.87** |
| ***ψ*(Slo+Ele+Can),*p*(Ele)** | **516.96** | **1.98** | **0.10** | **0.37** | **6** | **504.96** |
| *ψ*(.),*p*(Ele) | 517.8 | 2.82 | 0.07 | 0.24 | 3 | 511.8 |
| *ψ*(Ele+Can),*p*(Ele) | 518.6 | 3.62 | 0.05 | 0.16 | 5 | 508.6 |
| *ψ*(Can),*p*(Ele) | 519.64 | 4.66 | 0.03 | 0.10 | 4 | 511.64 |

Appendix S5. Estimates of Akaike weights for covariates that were hypothesized to influence the site use of Chinese muntjac (CM) and black muntjac (BM).

|  | model | AICw-Can | AICw-Slo | AICw-Ele |
| --- | --- | --- | --- | --- |
|  | *ψ*(Ele+Can),*p*(Slo) | 0.21 |  | 0.21 |
| CM | *ψ*(Can),*p*(Slo) | 0.19 |  |  |
|  | *ψ*(Ele),*p*(Slo) |  |  | 0.14 |
|  | *ψ*(Slo+Ele+Can),*p*(Slo) | 0.13 | 0.13 | 0.13 |
|  | *ψ*(Slo+Can),*p*(Slo) | 0.10 | 0.10 |  |
|  | *ψ*(.),*p*(Slo) |  |  |  |
|  | *ψ*(Slo+Ele),*p*(Slo) |  | 0.08 | 0.08 |
|  | *ψ*(Slo),*p*(Slo) |  | 0.05 |  |
| Summed Akaike weights |  | 0.63 | 0.37 | 0.56 |
| BM | *ψ*(Slo),*p*(Ele) |  | 0.27 |  |
|  | *ψ*(Slo+Ele),*p*(Ele) |  | 0.26 | 0.26 |
|  | *ψ*(Ele),*p*(Ele) |  |  | 0.12 |
|  | *ψ*(Slo+Can),*p*(Ele) | 0.11 | 0.11 |  |
|  | *ψ*(Slo+Ele+Can),*p*(Ele) | 0.10 | 0.10 | 0.10 |
|  | *ψ*(.),*p*(Ele) |  |  |  |
|  | *ψ*(Ele+Can),*p*(Ele) | 0.05 |  | 0.05 |
|  | *ψ*(Can),*p*(Ele) | 0.03 |  |  |
| Summed Akaike weights |  | 0.28 | 0.75 | 0.52 |
